# Genome-Wide Associations of Chlorophyll Fluorescence OJIP Transient Parameters Connected With Soil Drought Response in Barley

**DOI:** 10.3389/fpls.2019.00078

**Published:** 2019-02-11

**Authors:** Marcin Rapacz, Magdalena Wójcik-Jagła, Anna Fiust, Hazem M. Kalaji, Janusz Kościelniak

**Affiliations:** ^1^Department of Plant Physiology, University of Agriculture of Krakow, Krakow, Poland; ^2^Department of Grasslands, Institute of Technology and Life Sciences (ITP), Raszyn, Poland; ^3^Department of Plant Physiology, Faculty of Agriculture and Biology, Warsaw University of Life Sciences (SGGW), Warsaw, Poland

**Keywords:** barley, candidate genes, chlorophyll fluorescence, DArTseq, drought, genome-wide association analysis, OJIP

## Abstract

One hundred and nine accessions of spring barley seedlings were phenotyped under soil drought conditions. Chlorophyll fluorescence induction (OJIP) parameters, leaf water content, relative turgidity, net assimilation rate (*P*_N_), and water use efficiency (WUE) of plants were measured. All the tested lines were genotyped by means of DArT sequencing (DArTseq) technology. For association mapping a 11,780 polymorphic DArTseq and 4,725 DArTseq SNP markers were used. Our results revealed dissimilar patterns of the relationships between OJIP-parameters under control and drought conditions. A high level of correlation between parameters characterizing Photosystem's II (PSII) energy trapping efficiency (F_v_/F_m_) and photochemical events downstream of PSII reaction center (e.g., Performance Index—PI_CSo_) was observed only in the case of drought-treated plants. Generally, OJIP parameters were correlated with leaf water content (less in control). This correlation was weaker with WUE, and absent with *P*_N_. Under drought stress, 6,252 genotype × phenotype associations, which passed false discovery rate (FDR) verification, were found between all the studied phenotypic characteristics (23, including 19 OJIP parameters) and 2,721 markers. On the other hand, only 282 associations passed FDR test in the control. They comprised 22 phenotypic parameters and 205 markers. Probing for gene annotations of sequences was performed for markers associated with F_v_/F_m_ for both drought and control, markers were associated with studied traits in both control and drought, as well as for markers associated with both OJIP and other physiological parameters in drought. Our work allowed us to conclude that drought treatment differentiates the studied lines through the revealing of relationships between water content and the damages to PSII reaction centers or different components of PSII energy transfer chain. Moreover, the former was not connected with net photosynthesis rate.

## Introduction

Chlorophyll fluorescence measurements, including studies of induction kinetics in dark adapted samples followed by OJIP analysis, have been commonly used for studies of plant reaction to abiotic stresses (Kalaji et al., 2016). These include nitrogen deficiency (Cetner et al., 2017; Kalaji et al., 2017; Samborska et al., 2018) as well as low (Fracheboud et al., 1999; Rapacz et al., 2015) and high (Pastenes and Horton, 1996; Azam et al., 2015) temperature or salinity (Dabrowski et al., 2016). Also water stresses, like waterlogging (Bertholdsson et al., 2015) and especially water deficit (Li et al., 2006; Guo et al., 2008; Poormohammad Kiani et al., 2008; Czyczyło-Mysza et al., 2011; Wang et al., 2012), were widely studied using chlorophyll fluorescence techniques.

OJIP analysis may indicate electron fluxes between different components upstream, inside and downstream of PSII, thus they may indicate different points of drought-induced damages. Indeed it was possible to observe that the parameters of OJIP can very precisely characterize the state of leaf hydration (Živčák et al., 2008; Goltsev et al., 2012). However, the possible effects of water deficit on chlorophyll fluorescence parameters do not appear to be a simple reflection of the reduced energy demand from the dark photosynthesis phase.

The problem of drought tolerance is complex in general. Even the physiological and agronomic definition of drought tolerance varies. The physiological tolerance to drought requires the plant to maintain its vitality so that it can produce a minimum quantity of seeds or simply survive, while agronomic tolerance requires maintaining an economically significant yield (Schafleitner et al., 2007). Leaf hydration, critical for physiological tolerance, is not necessarily associated with drought tolerance in the agronomic sense, where the ability to maintain relatively high carboxylation is crucial for maintaining high productivity under drought (Blum, 2005; Ruggiero et al., 2017).

In our previous studies on tolerance to moderate water deficit for barley accessions with high carboxylation rates in drought under Middle-European conditions, we showed higher yielding potential in a dry environment (Rapacz et al., 2010; Wójcik-Jagła et al., 2012). However, no correlation between leaf hydration and net assimilation rate in drought was observed, considering both the lack of phenotypic correlations and the absence of common quantitative trait loci (QTLs) (Rapacz et al., 2010; Wójcik-Jagła et al., 2013). On the other hand, photosynthetic capacity in drought was clearly related to the expression level of the dehydrine encoding gene *HVA1* (Rapacz et al., 2010; Wójcik-Jagła et al., 2012). This complex relationship was probably connected to the proposed mechanism of the induction of *HVA1* expression in drought, which is triggered by intact changes of turgor in mesophyll cells sensed by the actin cytoskeleton (Wójcik-Jagła et al., 2012; Sniegowska-Swierk et al., 2015, 2016). To summarize, these studies showed that in barley, high photosynthetic activity can be maintained during moderate drought if the plants effectively protect the cells from dehydration irrespective of maintaining high turgidity at the cost of closing the stomata.

The basic effect of drought on photosynthesis is connected with stomatal limitation of carboxylation, where stomata closure results in a simultaneous decrease in water loss and CO_2_ uptake, which is the most challenging problem for breeding drought-tolerant cultivars (Lawson and Blatt, 2014; Flexas, 2016). Unfortunately, a higher water use efficiency (WUE) value results in lower stomatal conductance, which is often linked to a decrease of photosynthetic capacity, reducing productivity (Lawson and Blatt, 2014; Ruggiero et al., 2017). The stomatal limitation of photosynthesis in drought is the main, but not the only, effect of water deficit on photosynthesis. Metabolic impairment connected with deficiency of Calvin cycle intermediates, known as non-stomatal limitation, is observed under moderate drought conditions (Flexas and Medrano, 2002; Perlikowski et al., 2014).

Photosynthetic light-phase processes, directly studied by chlorophyll-fluorescence, are not considered as primary damage sites to photosynthetic apparatuses under water deficit (Kaiser, 1987; Cornic and Massacci, 1996). They are susceptible to secondary damage connected with photoinhibition, which occurs when the amount of energy absorbed by photosynthetic antennas exceeds the energy requirements of the dark photosynthesis phase (Sanda et al., 2011). In drought, this demand decreases mainly due to the stomata closing and, consequently, reactive oxygen species (ROS) production increases. Enhanced production of ROS in chloroplasts and the activation of hydrolytic enzymes in drought initiate damage of proteins, nucleic acids, and membrane lipids (Mittler, 2002). As a consequence, the photosynthetic electron transport chain is disturbed, but ROS are simultaneously signals necessary for maintaining redox homeostasis (Foyer and Shigeoka, 2011). Therefore, moderate drought can induce some adaptation of PSII observed as increasing energy fluxes for energy trapping and electron transfer (Kosmala et al., 2012). Similar reactions are also observed under heat or light excess conditions (Oukarroum et al., 2009; Pospíšil, 2016).

Decreased PSII activity under drought conditions is connected with cell membrane damage triggered by ROS and by disturbed lipid metabolism (Benhassaine-Kesri et al., 2002; Gallé and Feller, 2007). Additionally, dehydration affects cell turgor, causing mechanical damage to cellular membranes (Wolfe and Bryant, 1999). It has been proven that, unlike drought-sensitive plants, tolerant plants can maintain the integration of cell membranes in drought (Yu and Li, 2014). This phenomenon is connected with adaptive modifications of lipid composition in cellular membranes observed during water deficit in tolerant plants. It implies plastid membrane alterations, which in consequence greatly influences preserving transmembrane protein functions and thus eventually photosystem activities (Quartacci et al., 1995, 2000; Gigon et al., 2004; Degenkolbe et al., 2012; Perlikowski et al., 2016).

Plant drought stress is connected with the accumulation of low-molecular weight particles of complex function such as proline. Proline, which has long been considered a biochemical indicator of drought tolerance, can play an antioxidative and membrane-stabilizing function (Van Rensburg et al., 1993; Hayat et al., 2012). During drought, proline may affect the photosynthetic apparatus indirectly, through the osmotic action conducive to the maintenance of RWC, but also directly through the stabilization of cell membranes and RuBisCO.

Also ABA, which is a major plant hormone involved in plants' drought response and is considered as another biochemical indicator of drought tolerance, can affect both photosynthesis and chlorophyll fluorescence in drought by increasing relative water content in leaves (Corrêa de Souza et al., 2013; Mehrotra et al., 2014).

Despite the very well proved linkage between electron flows in PSII and leaf relative water content, the relationship between the latter and drought tolerance is still unclear. Therefore, we tried in this experiment to verify the hypothesis that the observed differences in chlorophyll fluorescence parameters in drought-treated plants are connected rather with leaf hydration and not with the efficiency of the carboxylation process. To confirm this hypothesis, in addition to the physiological tools, such as direct measurements of chlorophyll fluorescence, water status and gas exchange, we also attempted to screen the barley genome for the regions associated with OJIP parameters, gas exchange and water status in drought. We also tried to identify some genes affecting these relationships to suggest a possible explanation for this rather unexpected observation.

## Materials and Methods

### Plant Materials

The experiments were performed on the population of 109 accessions of barley consisting of doubled haploid-derived lines and F8 generation breeding materials from two breeding companies in Poland: HR Strzelce Ltd. group IHAR (Strzelce) and HR Danko sp. z o.o. (Choryń). The accessions were preselected by drought tolerance and targeted MAS, as described in detail elsewhere (Wójcik-Jagła et al., 2018). The complete list of accessions is available as Supplemental Material (Supplemental Table S1).

### Plant Growth and Drought Treatment

Twelve seeds of each accession were sown in a pot (5 dm^3^) in a mixture of universal garden soil substrate (Ekoziem, Jurków, Poland) and sand (1:1, v:v). To allow subsequent measurements only at midday, 10 accessions per day were sown. Pots were randomized in a growth chamber set to 25°C/17°C (day/night). A photoperiod of 14/10 h was applied and the light (400 μmol m^−2^ s^−1^) was provided by high-pressure sodium (HPS) lamps (SON-T+ AGRO, Philips, Brussels, Belgium). The plants were watered to 10% of the soil dry weight (equal to 3 pF—logarithm from soil matrix potential, based on soil water retention curve), which was checked daily by weighing. Once a week, fertilization with Florovit multi-component fertilizer (Inco, Góra Kalwaria, Poland) was performed. Soil drought was applied at 4th leaf stage by decreasing watering to reach 4.0 pF (3.5% of the soil dry weight). At this pF, visual symptoms of reduced turgidity were visible in all accessions. The drought treatment lasted 10 days after reaching the required pF and soil moisture retention curve. Phenotypic measurements were taken 1 day before starting the soil water content decrease (control) and at the last day of drought treatment.

### Phenotyping

The measurements and samplings were always performed between 11 a.m. and 2 p.m. because of diurnal changes expected for gas exchange and chlorophyll fluorescence.

Chlorophyll fluorescence measurements were taken in the middle part of the second leaf of 10 plants per accession. Measurements of the fast chlorophyll fluorescence induction kinetics were taken with Handy PEA (Hansatech, Kings Lynn, UK), with saturating flash intensity ca. 3,000 μmol m^−2^ s^−1^ and measurement time 1 s. Measurements of photo-induced chlorophyll fluorescence transients were used to calculate characteristics of the light phase of photosynthesis according to the OJIP algorithm (Strasser et al., 2004; Gururani et al., 2015). Measured and calculated parameters are listed in Table 1 and detailed calculation formulas can be found elsewhere (Gururani et al., 2015; Rapacz et al., 2015).

**Table 1:** OJIP-test parameters calculated in the study.

**SPECIFIC ENERGY FLUXES (PER PRIMARY ELECTRON ACCEPTOR IN PHOTOSYSTEM II (PSII) – QUINONE A (Q**_****A****_**)-REDUCING PSII REACTION CENTER—RC)**	
ABS/RC	Absorption flux (of antenna chlorophylls) per RC
TR_o_/RC	Trapping flux (leading to Q_A_ reduction) per RC
ET_o_/RC	Electron transport flux (further than Q$$$) per RC
RE_o_/RC	Electron flux reducing end electron acceptors at the photosystem I (PSI) acceptor side per RC
DI_o_/RC	Dissipated energy flux per RC (at *t* = 0)
**QUANTUM YIELDS AND EFFICIENCIES**	
F_v_/F_m_ (ϕ_Po_)	Maximum quantum yield of primary photochemistry (at *t* = 0)
Ψ_o_	Probability/efficiency (at *t* = 0) that a trapped exciton moves an electron into the electron transport chain beyond Q_A_
ϕ_Eo_	Quantum yield of electron transport (at *t* = 0)
ϕ_Ro_	Quantum yield of reduction of end electron acceptors at the PSI acceptor side (RE)
**PHENOMENOLOGICAL FLUXES**	
ABS/CS	Phenomenological absorption flux per excited cross-section (CS)
TR_o_/CS	Phenomenological trapping flux per excited CS
ET_o_/CS	Phenomenological electron transport flux per excited CS
DI_o_/CS	Phenomenological dissipated energy flux per excited CS
RC/CS_o_	Density of Reaction Centers - RCs (Q$$$ reducing PSII reaction) at *t* = 0
RC/CS_m_	Density of Reaction Centers - RCs (Q$$$ reducing PSII reaction) at t_max_ (time to reach maximum fluorescence F_m_)
**PERFORMANCE INDEXES**	
PI_x_	Performance indexes (potential) for energy conservation from exciton to the reduction of intersystem electron acceptors:
PI_ABS_	calculated on the basis of absorption
PI_CSo_	calculated on the basis of density of Q_A_ - reducing PSII reaction centers at *t* = 0
PI_CSm_	calculated on the basis of density of Q_A_ - reducing PSII reaction centers at t_max_
PI_total_	Performance index (potential) for energy conservation from exciton to the reduction of PSI end acceptors

Plant gas exchange parameters were also measured in the middle part of the second barley leaves with a Ciras-3 infrared gas analyzer (PP Systems, MA, United States) equipped with a Universal Leaf Chamber (PLC6). The controlled conditions of the measurements were as follows: CO_2_ concentration of 400 μmol mol^−1^, relative humidity of 30%, irradiance of 500 μmol m^−2^ s^−1^, and leaf temperature of 25°C. The measurements were taken in 10 plants per accession. From the measured gas exchange parameters, two were used for further calculations: net CO_2_ assimilation rate (*P*_N_) and stomatal conductance (*g*_s_). Both were used for calculation of water use efficiency (WUE): WUEi = *P*_N_/*g*_s_ (Flexas, 2016).

Measurements of leaf water status were performed on eight randomly selected first (the oldest) leaves of each accession. After cutting, the leaves were weighed (fresh weight; FW), placed in 25-ml closed tubes filled with water, and shaken in darkness. After 24 h the turgor weight (TW) was determined and the leaves were then dried for 48 h in paper envelopes at a temperature of 70°C in the laboratory dryer (Lumel, Zielona Góra, Poland). The dry weight (DW) was then determined. Relative water content (RWC) or relative turgidity was calculated as RWC [%] = (FW – DW)/(TW – DW) × 100% and water content (WC) as WC [g H_2_O/ g DW] = (FW – DW)/DW (Barrs, 1968).

### Genotyping and Genome Wide Associations

One hundred and nine selected spring barley lines were genotyped using DArT sequencing technology (DArTseq) (https://www.diversityarrays.com/products-and-services/applications/), as previously described (Wójcik-Jagła et al., 2018). The genotyping resulted in 15,828 specific DArTseqs and 7,829 SNPs. The input data for the population's structure and association analysis were only markers with Polymorphism Information Content (PIC) > 18% (11,780 DArTseqs and 4,725 SNPs). Association analysis included the entire genome (GWAS). The analysis of the population structure was carried out using the STRUCTURE v. 2.3.4 software (Stanford University, California), (Pritchard et al., 2000). The following admixture model was selected: 10,000 cycles and 1,000 repetitions per cycle. The test was carried out 10 times for six possible subpopulations (*K* = 1–6). The true value of K parameter was determined in the manner described by Evanno et al. (2005). The marker-trait associations were determined using the TASSEL program with TASSEL software (Ithaca, New York, NY, United States) (Bradbury et al., 2007), as demonstrated previously (Wójcik-Jagła et al., 2018).

A minimum allele frequency of 0.05 was required for the genotype data. The degree of kinship of the population used in this study was strictly controlled, therefore we did not perform any correction for kinship. The general linear model (GLM) was used to determine the associations, finding the ordinary least squares solution for each marker-trait association (Bradbury et al., 2007). The probability level threshold was 0.001. The additive model was used for data analysis. Obtained probability values were corrected using the false discovery rate (FDR) (Benjamini and Hochberg, 1995) and Bonferroni correction (Dunn, 1958).

### Annotations for Markers

The homology of selected barley DarTseq/SNP marker sequences with potential functional genes or proteins was confirmed with the BLASTn algorithm at http://blast.ncbi.nlm.nih.gov (Altschul et al., 1990) using discontiguous megablast as a selection program. The characterization of identified homologs with the highest similarity estimated on Qc (query cover) and Id (sequence identity) parameters was done with GeneBank datasets at https://www.ncbi.nlm.nih.gov/genbank/ (Benson et al., 2012). The identification of potential genes in the sequence of the barley genome was conducted by the ViroBLAST server at http://webblast.ipk-gatersleben.de (Deng et al., 2007) on a base from the barley high-confidence genes database (HC_genes_CDS_seq_2012). Moreover, functional annotations and gene ontology of selected sequences were confirmed by the sets from the UniProt and InterPro databases, located at http://www.uniprot.org/ (Magrane and Uniprot Consortium, 2011) and https://www.ebi.ac.uk/interpro/search/sequence-search (Mitchell et al., 2015), respectively. Conserved domain identification in marker sequences was confirmed by the NCBI Conserved Domain Database (CDD), located at https://www.ncbi.nlm.nih.gov/Structure/cdd/cdd.shtml (Marchler-Bauer et al., 2015).

### Statistical Treatment of Phenotype Data

Data was processed using Statistica 13.1PL software (Statsoft, Tulsa, OK). Statistical significance of the drought effect on phenotypic data was checked by means of multifactor ANOVA in a General Linear Model (GLM) with accession and environment as factors. Normal distribution of the data was confirmed with histograms and Shapiro-Wilk testing. Pearson's correlation coefficients between phenotypic data were calculated based on the mean values for the accession. Principal component analyses (PCA) were performed by eigenvalue decomposition of a data correlation matrix.

## Results

Drought significantly affected all the physiological parameters studied in this study. With exception to ET_o_/RC, DI_o_/RC, ϕ_Eo_ and Ψ_o_, the values of all parameters decreased under drought conditions, including an over 4-fold reduction of *P*_N_ and 2-fold reduction in WC (Table 2). Among OJIP parameters, the relative magnitude of change was the highest in the case of TR_o_/CS and RC/CS_m_, which indicates that the drought mainly limited the number of active PSII reaction centers and thus the amount of trapped energy per leaf cross-section. All the changes were highly statistically significant, with exception of DI_o_/RC, which was significant at *P* = 0.05 (Supplemental Table S1). Net assimilation rate (*P*_N_) in both environments was not correlated with other physiological parameters with the exception of WUE, which was measured with the same method (Table 2). WC and RWC were correlated with WUE in drought only (Table 2). In well-watered plants, only some correlations between OJIP parameters and WC and RWC were observed (Table 2). This included negative correlations with phenomenological energy fluxes at different stages of electron transport (…/CS), RC/CS_m_ and positive with F_v_/F_m_. The values of correlation coefficients were always under 0.5. Under drought stress, physiological parameters related to leaf water status directly (WC, RWC) or indirectly (WUE) were correlated with more OJIP parameters than in the control, but the values of correlation coefficients were still rather low. They were correlated with OJIP parameters related to PSII efficiency (e.g., F_v_/F_m_, Ψ_o_, ϕ_Eo_, PI_ABS_, PI_CSo_, PI_CSm_) and activity of single PSII reaction centers (…/RC) (Table 2). It may be suggested that the main reason for drought-induced changes in OJIP parameters is direct damage to Photosystem II (PSII) reaction centers or different components of the energy transfer chain in PSII. This may be additionally confirmed by the fact that the parameter characterizing energy trapping efficiency in PSII reaction centers (F_v_/F_m_), which is affected by PSII dysfunction, was highly correlated with the majority of OJIP parameters, including performance indexes (PI_ABS_, PI_CSo_, PI_CSm_) only in drought and not under control conditions.

**Table 2:** Means, standard deviations and correlation matrix between studied phenotypic characteristics measured under control (shaded) and after 10 days of drought treatment (4 pF, no shading) conditions.

**Trait**	**Mean control**	**St. dev. control**	**Mean drought**	**St. dev. drought**	**F_**v**_/F_**m**_**	**ABS/RC**	**Ψ_o_**	**ϕ_Eo_**	**PI_**CSo**_**	**PI_**CSm**_**	**PI_**ABS**_**	**ABS/CS**	**TR_**o**_/CS**	**ET_**o**_/CS**	**DI_**o**_/CS**	**RC/CS_**o**_**	**RC/CS_**m**_**	**ET_**o**_/RC**	**TR_**o**_/RC**	**DI_**o**_/RC**	**PIt_**otal**_**	**RE_**o**_/RC**	**ϕ_Ro_**	**P_**n**_**	**WUE**	**WC**	**RWC**
F_v_/F_m_	0.805	0.006	0.783	0.027		–0.87	0.62	0.83	0.80	0.82	0.79	–0.30	0.35	0.60	–0.92	0.67	0.88	0.09	–0.50	–0.95	0.43	–0.36	−0.01	−0.08	0.14	0.42	0.22
ABS/RC	2.451	0.152	2.304	0.238	−0.12		–0.70	–0.83	–0.86	–0.85	–0.87	0.41	−0.15	–0.52	0.85	–0.81	–0.88	0.17	0.82	0.92	–0.49	0.44	0.03	−0.08	–0.31	–0.29	−0.04
Ψ_o_	0.457	0.030	0.524	0.030	0.08	-0.56		0.95	0.86	0.79	0.83	−0.13	0.26	0.81	–0.53	0.64	0.65	0.43	–0.55	–0.66	0.84	0.10	0.49	0.17	0.20	0.29	0.12
ϕ_Eo_	0.368	0.025	0.411	0.032	0.18	-0.56	0.99		0.94	0.91	0.92	–0.24	0.29	0.79	–0.74	0.71	0.82	0.31	–0.61	–0.81	0.76	−0.11	0.31	0.08	0.20	0.38	0.18
PI_CSo_	562.8	99.86	507.1	112.2	0.19	-0.77	0.90	0.91		0.99	0.99	–0.27	0.25	0.72	–0.73	0.82	0.92	0.05	–0.76	–0.75	0.72	–0.28	0.16	0.05	0.27	0.36	0.08
PI_CSm_	2899	538.3	2460	666.4	0.34	-0.76	0.87	0.90	0.99		0.99	–0.32	0.21	0.65	–0.77	0.79	0.94	−0.01	–0.75	–0.74	0.67	–0.34	0.08	0.03	0.26	0.40	0.11
PI_ABS_	1.444	0.255	1.910	0.451	0.30	-0.78	0.92	0.93	0.96	0.97		–0.42	0.09	0.60	–0.78	0.75	0.90	−0.03	–0.80	–0.74	0.68	–0.36	0.08	0.08	0.29	0.37	0.11
ABS/CS	390.1	18.85	267.5	15.35	–0.40	0.00	−0.05	−0.09	0.16	0.09	−0.12		0.78	0.39	0.65	0.12	–0.19	0.43	0.53	0.24	0.09	0.69	0.54	−0.07	−0.10	−0.18	–0.24
TR_o_/CS	314.2	14.45	209.1	11.70	–0.27	−0.02	−0.04	−0.06	0.19	0.14	−0.08	0.99		0.77	0.03	0.55	0.38	0.48	0.21	–0.36	0.35	0.43	0.50	−0.15	−0.02	0.09	−0.09
ET_o_/CS	143.5	11.45	109.9	9.006	−0.09	-0.47	0.81	0.79	0.86	0.81	0.72	0.54	0.55		–0.31	0.75	0.65	0.57	–0.24	–0.62	0.76	0.32	0.62	0.01	0.12	0.24	0.02
DI_o_/CS	75.92	4.956	58.33	9.553	-0.73	0.05	−0.07	−0.14	0.03	−0.08	-0.22	0.92	0.85	0.44		–0.48	–0.77	0.11	0.60	0.84	–0.28	0.58	0.25	0.07	−0.14	–0.40	–0.26
RC/CS_o_	137.0	9.691	99.97	7.395	−0.15	-0.74	0.39	0.37	0.69	0.63	0.52	0.64	0.65	0.70	0.54		0.89	−0.11	–0.70	–0.71	0.58	−0.18	0.20	0.01	0.33	0.17	−0.18
RC/CS_m_	704.7	50.93	473.9	64.76	0.25	-0.78	0.42	0.44	0.75	0.76	0.63	0.47	0.53	0.65	0.24	0.92		−0.13	–0.72	–0.81	0.53	–0.40	−0.01	−0.06	0.27	0.36	0.03
ET_o_/RC	0.900	0.055	0.934	0.054	0.07	0.41	0.52	0.52	0.20	0.20	0.22	−0.10	−0.09	0.39	−0.10	-0.35	-0.32		0.52	−0.10	0.42	0.72	0.71	0.02	–0.22	0.15	0.26
TR_o_/RC	1.974	0.122	1.790	0.112	−0.01	0.99	-0.55	-0.55	-0.76	-0.73	-0.75	−0.05	−0.05	-0.49	−0.03	-0.77	-0.75	0.42		0.52	–0.39	0.58	0.20	−0.14	–0.38	−0.13	0.12
DI_o_/RC	0.477	0.034	0.514	0.159	–0.51	0.92	–0.52	–0.56	–0.75	–0.80	–0.80	0.16	0.09	-0.38	0.34	–0.59	–0.78	0.32	0.86		–0.47	0.25	−0.11	−0.01	–0.19	–0.34	−0.14
PI_total_	1.203	0.300	0.874	0.199	0.02	-0.53	0.47	0.46	0.55	0.53	0.52	0.13	0.14	0.47	0.09	0.49	0.49	−0.04	–0.53	–0.47		0.40	0.76	0.14	0.28	0.10	−0.09
RE_o_/RC	0.405	0.048	0.294	0.061	–0.20	0.22	0.06	0.04	−0.03	−0.06	−0.10	0.22	0.20	0.16	0.25	−0.01	−0.09	0.27	0.20	0.28	0.68		0.89	0.12	−0.04	–0.21	−0.14
ϕ_Ro_	0.166	0.020	0.129	0.019	−0.13	-0.29	0.35	0.33	0.37	0.33	0.31	0.21	0.20	0.40	0.21	0.36	0.31	0.06	–0.31	–0.20	0.95	0.86		0.15	0.12	−0.12	−0.16
*P*_N_	22.25	2.114	5.312	1.601	0.02	-0.20	0.04	0.04	0.14	0.13	0.10	0.13	0.14	0.11	0.09	0.23	0.23	−0.16	-0.20	−0.18	−0.02	−0.16	−0.05		0.46	−0.08	−0.02
WUE	10.39	0.560	6.773	3.515	0.05	0.04	-0.21	-0.20	−0.11	−0.09	−0.14	0.14	0.15	−0.09	0.08	0.05	0.07	−0.18	0.05	0.02	−0.13	−0.12	−0.13	0.42		–0.21	–0.25
WC	6.575	0.760	3.280	0.764	0.32	0.01	0.00	0.04	−0.05	0.00	0.08	–0.47	–0.45	–0.26	–0.49	–0.29	−0.16	0.05	0.05	−0.12	−0.05	−0.11	−0.10	−0.14	−0.12		0.68
RWC	85.75	2.458	49.17	7.395	0.33	0.05	−0.08	−0.04	−0.07	−0.02	0.01	-0.31	-0.27	-0.23	-0.37	-0.23	−0.09	−0.01	0.08	−0.09	−0.18	-0.21	-0.23	−0.08	−0.04	0.64	

*Linear correlation coefficients (Pearson's) indicated in red are statistically significant, with p < 0.05000. N = 109. All the means are statistically significantly different between control and drought treatment for p < 0.001, with exception of DI_o_/RC (p < 0.05)*.

**Figure 1:**
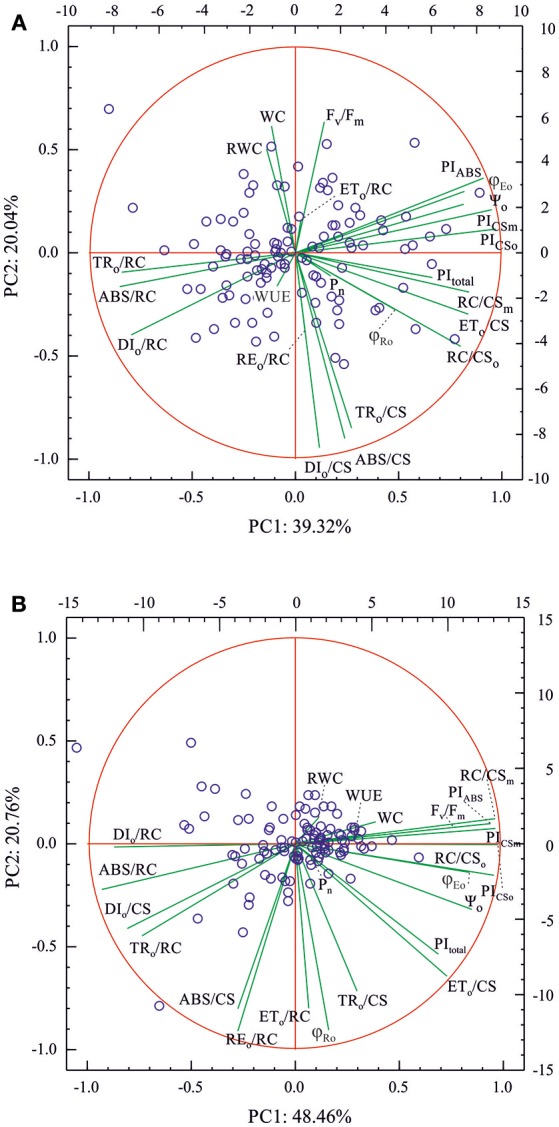
Biplots of principal component analysis (PCA) for OJIP parameters, leaf water relations (WC, RWC), WUE and *P*_N_ (variables) measured in 109 barley accessions (cases) in **(A)** well-watering conditions (control) and **(B)** in drought. Left y and bottom x axes refer to variables and right y and top x axes for the cases, respectively.

The various patterns of the relationships between different OJIP parameters, leaf water relations and P_N_ in the control and drought-treated plants were also shown by principal component analysis (PCA) (Figure 1 and Supplementary Table S2). In both control and drought-treated plants, the first two principal components described much more than 50% of the total variation (60 and 70% for the control and drought-treated plants, respectively) and did not reveal physiological subpopulations of the studied accessions. Only some outliers, different for both environments, were present. In both environments, WC and RWC (in drought also WUE) discriminated the accessions in a similar way to that of F_v_/F_m_ and DI_o_/CS (consider that higher values of DIo/CS indicated low energy trapping efficiency in PSII reaction centers as lower values of F_v_/F_m_ do). ET_o_/RC had a very similar but weaker effect on the accession coordinates to those observed for WC and RWC in the control conditions. Under drought conditions, the accession discrimination effects of RWC, WC, and WUE were similar to those of all the performance indexes for energy conservation from exciton to the reduction of intersystem electron acceptors (PI_ABS_, PI_CSo_, PI_CSm_) and the numbers of active PSII reaction centers (RC/CS_o_, RC/CS_m_). In both environments, net assimilation rate (*P*_N_) had a small effect on the differences between accessions, and it was similar to those of OJIP parameters describing efficiency of electron transport in PSII and at the PSI end acceptors side (as ET_o_/CS, ϕ_Ro_, or PI_total_). On the other hand, the direction of accession discrimination for *P*_N_ was similar to that of the number of active PSII reaction centers (RC/CS_o_, RC/CS_m_) in well-watered plants only.

In the control, 282 associations between phenotypes and markers, which passed false discovery rate (FDR) verification were found (Supplementary Table S3). They comprised 22 phenotypic parameters and 205 markers. On the other hand, under drought stress 6,252 genotype × phenotype associations passed the FDR test (Supplementary Table S4). These associations were found between all the studied phenotypic characteristics (23, including 19 OJIP parameters) and 2,721 markers.

F_v_/F_m_, describing the efficiency of energy trapping in PSII reaction centers, is the most commonly analyzed chlorophyll fluorescence parameter. In our study, it showed a similar relation with leaf water status in the control and drought, but it was correlated with efficiencies of further steps in photosynthetic energy transfer only in drought. Thus, it was chosen for testing for gene annotations of associated marker sequences (Tables 3, 4 and Supplementary Tables S5, S6). In well-watered plants, F_v_/F_m_ values were associated with 20 markers, including 5 SNPs. Among them, 5 sequences were annotated to protein sequences in the UniProt database and only one with GO annotation for barley protein (proteolytic activity) (Table 3). On the other hand, in drought-treated plants F_v_/F_m_ values were associated with 40 markers, among them 8 (including 5 SNPs) have GO annotations for proteins (Table 4). Four of them may possess protein kinase activity, three protein binding activity and one sequence (3432879) has 100% homology with wheat's 3-ketoacyl-CoA synthase, a transferase involved in the fatty acid biosynthesis process. Interestingly, another marker (3262177) associated with OJIP parameters in both environments shows 100% homology with the sequence of genes encoding the same enzyme in barley (Table 5). Thus, this protein may be important for energy conversion and transfer efficiency in thylakoids regardless of drought.

**Table 3:** Gene annotations of DArTseq and SNP markers associated with energy trapping efficiency in PSII (F_v_/F_m_) in well-watered barley (control).

**Marker**	**Chromosome location**	**Number of genotypes and subpopulation affinity**	**UniProt**	**GO annotations**	**CD**
3260252	bowman_contig_880154|6|17.71|15111880	53: 11(1), 7 (1/2), 19 (2), 16 (3)	A0A287U3L0 Uncharacterized protein (*Hordeum vulgare* subsp. *vulgare*) 84.6%	Biological Process: GO:0006508 proteolysis Molecular Function: GO:0004185 serine-type carboxypeptidase activity Cellular Component: -	–
3268966	morex_contig_222738|5|29.10|18794680	69: 26(1), 5(1/2), 22(2), 16(3)	M8AIA5 Disease resistance protein RPM1 (*Triticum urartu*) 80%	Biological Process: - Molecular Function: GO:0043531 ADP binding Cellular Component: -	–
3269029	bowman_contig_128910|5|92.99|460586840	24: 18(1), 4(1/2), 2(2), 0(3)	F2E8H5 Predicted protein (*Hordeum vulgare* subsp. *vulgare*) 100%	Biological Process: - Molecular Function: - Cellular Component: -	–
3256262*	morex_contig_1559060|5|39.62|32903400	97: 33(1), 14(1/2), 30(2), 20(3)	A0A287QCK5 Uncharacterized protein (*Hordeum vulgare* subsp. *vulgare*) 100%	Biological Process: - Molecular Function: - Cellular Component: -	–
3255896*	morex_contig_1592149|5|41.74|42555560	96: 33(1), 14(1/2), 29(2), 20(3)	A0A0Q3HCC9 Uncharacterized protein (*Brachypodium distachyon*) 62.5%	Biological Process: - Molecular Function: - Cellular Component: -	–

*SNPs are asterisked. Exact chromosome location of the marker with gene annotation, number of genotypes carrying allele “1” or “1” and “2” (SNP markers) and their subpopulation affinity (by STRUCTURE), regions of local similarity of marker sequence described by UniProt (protein ID, species, percentage of similarity [%]), Gene Ontology (GO) assigned by InterPro database, CD—conserved domain identified by NCBI Conserved Domain Database (CDD). ‘-' non-predicted. Only markers with UniProt annotations are presented. For the whole dataset see Supplementary Table S5*.

**Table 4:** Gene annotations of DArTseq and SNP markers associated with energy trapping efficiency in PSII (F_v_/F_m_) in barley plants under drought.

**Marker**	**Chromosome location**	**Number of genotypes and subpopulation affinity**	**UniProt**	**GO annotations**	**CD**
3432879	morex_contig_2552250|7|29.82|36768480	21: 5(1), 0(1/2), 2(2), 14(3)	A0A287VP74 Uncharacterized protein (*Hordeum vulgare* subsp. *vulgare*), 100%, A0A1D6BXJ6 3-ketoacyl-CoA synthase (Triticum aestivum), 100%	Biological Process: GO:0006633 fatty acid biosynthetic process, GO:0008152 metabolic process Molecular Function: GO:0003824 catalytic activity, GO:0016747 transferase activity, transferring acyl groups other than amino-acyl groups Cellular Component: GO:0016020 membrane	cl09938 cond_enzymes super family
3661735	morex_contig_1559786|6|108.29|524059960	14: 12(1), 2(1/2), 0(2), 0(3)	A0A287V3B9 Predicted protein (*Hordeum vulgare* subsp. *vulgare*), 100%	Biological Process: -Molecular Function: GO:0005515 protein binding Cellular component: -	-
3986939	-	26: 20(1), 2(1/2), 4(2), 0(3)	A0A1D5YYY1 Uncharacterized protein (*Triticum aestivum*), 100%	Biological Process: -Molecular Function: GO:0005515 protein binding Cellular component: -	-
100002035*	bowman_contig_81449|5|111.32|483596280	14: 10(1), 2(1/2), 2(2), 0(3)	A0A287S550 Uncharacterized protein (*Hordeum vulgare* subsp. *vulgare*), 92.3%	Biological Process: GO:0006468 protein phosphorylation, GO:0048544 recognition of pollen Molecular Function: GO:0004672 protein kinase activity, GO:0005524 ATP binding Cellular component: -	-
3266819*	barke_contig_1800369|5|109.65|481385360	14: 10(1), 2(1/2), 2(2), 0(3)	A0A1D5Z2I8 Uncharacterized protein (*Triticum aestivum*), 95.5%	Biological Process: -Molecular Function: GO:0005515 protein binding Cellular component: -	-
3397479*	bowman_contig_64191|5|110.93|481616000	14: 10(1), 2(1/2), 2(2), 0(3)	A0A287E1P0 Uncharacterized protein (*Hordeum vulgare* subsp. *vulgare*), 100%	Biological Process: GO:0006468 protein phosphorylation, GO:0048544 recognition of pollen Molecular Function: GO:0004672 protein kinase activity, GO:0005524 ATP binding Cellular component: -	-
4172735*	bowman_contig_64191|5|110.93|481616000	14: 10(1), 2(1/2), 2(2), 0(3)	A0A287E1P0 Uncharacterized protein (*Hordeum vulgare* subsp. *vulgare*), 100%	Biological Process: GO:0006468 protein phosphorylation, GO:0048544 recognition of pollen Molecular Function: GO:0004672 protein kinase activity, GO:0005524 ATP binding Cellular component: -	cl00112 PAN_APPLE super family
5249526*	bowman_contig_81449|5|111.32|483596280	80: 18(1), 13(1/2), 29(2), 20(3)	A0A287S550 Uncharacterized protein (*Hordeum vulgare* subsp. *vulgare*), 100% M8C7I9 Putative serine/threonine-protein kinase receptor (*Aegilops tauschii*), 81%	Biological Process: GO:0006468 protein phosphorylation, GO:0048544 recognition of pollen Molecular Function: GO:0004672 protein kinase activity, GO:0005524 ATP binding Cellular component: -	-

*SNPs are asterisked. Exact chromosome location of the marker with gene annotation, ‘-‘unknown, number of genotypes carrying allele “1” or “1” and “2” (SNP markers) and their subpopulation affinity (by STRUCTURE), regions of local similarity of marker sequence described by UniProt (protein ID, species, percentage of similarity [%]), Gene Ontology (GO) assigned by InterPro database, CD—conserved domain identified by NCBI Conserved Domain Database (CDD). ‘-' non-predicted. Only markers with UniProt annotations are presented. For the whole dataset see Supplementary Table S6*.

**Table 5:** Gene annotations of DArTseq and SNP markers associated with physiological parameters in both well-watered (control) and drought-treated barley plants.

**PP control**	**PP drought**	**Marker**	**Chromosome location**	**Number of genotypes and subpopulation affinity**	**UniProt**	**GO annotations**	**CD**
RE_o_/RC	Ψ_o_ ϕ_Eo_ PI_CSo_ ET_o_/CS	3262177	bowman_contig_881524|4|3.47|6723280	41: 19(1), 10(1/2), 12(2), 0(3)	A0A287ECA3 3-ketoacyl-CoA synthase (*Hordeum vulgare* subsp. *vulgare*), 100%	Biological Process: GO:0006633 fatty acid biosynthetic process, GO:0008152 metabolic process Molecular Function: GO:0003824 catalytic activity, GO:0016747 transferase activity, transferring acyl groups other than amino-acyl groups Cellular Component: GO:0016020 membrane	cl09938 cond_enzymes super family
RE_o_/RC	RC/CS_o_	3987061	bowman_contig_63772|4|51.20|189020640	83: 24(1), 13(1/2), 30(2), 16(3)	-	Biological Process: GO:0006696 ergosterol biosynthetic process, GO:0009058 biosynthetic process Molecular Function: GO:0004310 farnesyl-diphosphate farnesyltransferase activity, GO:0016740 transferase activity, GO:0016765 transferase activity, transferring alkyl or aryl (other than methyl) groups, GO:0051996 squalene synthase activity Cellular Component: -	-
RC/CS_m_ PI_CSm_	F_v_/F_m_	3268966	morex_contig_222738|5|29.10|18794680	69: 26(1), 5(1/2), 22(2), 16(3)	M8AIA5 Disease resistance protein RPM1 (*Triticum urartu*), 80%	Biological Process: - Molecular Function: GO:0043531 ADP binding Cellular Component: -	-
RE_o_/RC	TR_o_/RC	3431571	bowman_contig_94592|4|50.85|98696560	20: 16(1), 0(1/2), 2(2), 2(3)	M8B1N6 Putative serpin-Z8 (*Aegilops tauschii*), 83.3%	Biological Process: - Molecular Function: GO:0043531 ADP binding Cellular Component: -	-
ET_o_/CS	PI_total_	4174101	bowman_contig_1990327|1|119.69|452947200	78: 30(1), 6(1/2), 23(2), 19(3)	M8BJ05 Speckle-type POZ protein-like protein A (*Aegilops tauschii*), 91.3%	Biological Process: - Molecular Function: GO:0005515 protein binding Cellular Component: -	-
ET_o_/CS	PI_total_	7232846	barke_contig_283132|1|119.33|449889360	22: 7(1), 7(1/2), 8(2), 0(3)	A0A287GMB7 Uncharacterized protein (*Hordeum vulgare* subsp. *vulgare*), 90.9%	Biological Process: - Molecular Function: - Cellular Component: -	-
DI_o_/CS F_v_/F_m_	WUE	3256262*	morex_contig_1559060|5|39.62|32903400	97: 33(1), 14(1/2), 30(2), 20(3)	A0A287QCK5 Uncharacterized protein (*Hordeum vulgare* subsp. *vulgare*), 100%	Biological Process: - Molecular Function: - Cellular Component: -	-
RE_o_/RC	WC	3255247*	morex_contig_45560|3|88.53|484326640	10: 2(1), 4(1/2), 4(2), 0(3)	-	Biological Process: GO:0006355 regulation of transcription, DNA-templated Molecular Function: GO:0003690 double-stranded DNA binding Cellular Component: -	-

*SNPs are asterisked. PP—physiological parameter. Exact chromosome location of the marker with gene annotation, number of genotypes carrying allele “1” or “1” and “2” (SNP markers) and their subpopulation affinity (by STRUCTURE), regions of local similarity of marker sequence described by UniProt (protein ID, species, percentage of similarity [%]), Gene Ontology (GO) assigned by InterPro database, CD—conserved domain identified by NCBI Conserved Domain Database (CDD). ‘-' non-predicted. Only markers with UniProt annotations are presented. For the whole dataset, see Supplementary Table S7*.

Forty-one markers were associated with phenotypic traits, both in control and drought (28 with annotations found) (Supplementary Table S7). Under control conditions, eight of these common markers, including the aforementioned 3262177, were associated with electron flux reducing end electron acceptors at the Photosystem I (PSI) acceptor side per reaction center (RC) (RE_o_/RC), while in drought-treated plants the same markers were associated with OJIP parameters characterizing upstream stages of the electron transport or even water content in leaves. Additionally, four further markers were associated with WUE in drought-treated plants while in the control, with some OJIP parameters. In eight cases, UniProt or/and GO annotations were identified for markers common for drought and well-watered plants (Table 5). With the exception of 3262177 and SNP 3256262 (putative DNA-binding transcription factor), other sequences were not fully characterized with respect to their function and/or with high sequence homology to barley proteins. In one case, GO molecular function of protein partially encoded by a marker indicated ergosterol biosynthesis (not synthesized in higher plants) and in other cases proteins with ADP or protein binding activities.

In the context of drought effect on plants, what is most interesting are the markers associated with physiological parameters belonging to at least two of the following three groups: gas exchange (WUE, *P*_N_), leaf water status (WC, RWC) and OJIP parameters (Table 6 and Supplementary Table S8). A total of 162 associations meeting these criteria were found (Supplementary Table S8). Among them, none of the *P*_N_-related sequences were found, which confirmed the very small relationship between net assimilation rate and both OJIP and leaf water status parameters in drought-treated barley. A total of 51 associations did not include OJIP parameters, and the associated sequences were common for RWC and WUE in 48 cases and in three cases for WC and RWC. Furthermore, 29 sequences associated with both WUE and RWC and additionally with OJIP parameters were found, which confirms a strong, authentic and not only statistical link between RWC and WUE, which are measured by completely different methods but were related to stomatal conductance. In any case, WC was associated with the same sequences as OJIP parameters. Among 112 associations common for OJIP parameters and WUE/RWC, all were related to the parameters connected with energy fluxes in single PSII reaction centers, mainly with TR_o_/RC and ABS/RC. In 25 cases, RWC/WUE shared the same associated markers with the number of active reaction centers (RC/CS_o_, RC/CS_m_), and in a further 9 cases also with at least one PSII performance index for energy conservation from exciton to the reduction of intersystem electron acceptors (PI_ABS_, PI_CSo_, PI_CSm_). Only one case association common for OJIP parameters and other physiological characteristics concerned a parameter describing phenomenological energy fluxes per leaf cross-section, and in no case was there a parameter describing photochemical activity at the Photosystem I (PSI) acceptors side.

**Table 6:** Gene annotations of DArTseq and SNP markers associated with at least two physiological traits in barley plants under drought with the exception of the markers associated only with chlorophyll fluorescence parameters.

**PP**	**Marker**	**Chromosome location**	**Number of genotypes and subpopulation affinity**	**UniProt**	**GO annotations**	**CD**
ET_o_/RC RWC TR_o_/RC WUE	3255929	morex_contig_234306|2|5.52|6729480	55: 13(1), 10(1/2), 18(2), 14(3)	A0A151TV76 (*Cajanus cajan*), Wall-associated receptor kinase-like 20 84,6%	Biological Process: GO:0006468 protein phosphorylation Molecular Function: GO:0004672 protein kinase activity, GO:0005524 ATP binding, GO:0030247 polysaccharide binding Cellular Component: -	-
ABS/RC ET_o_/RC TR_o_/RC WUE	3256062	barke_contig_370440|2|5.38|6729480	72: 32(1), 6(1/2), 23(2), 11(3)	F2DHH6 Predicted protein (*Hordeum vulgare* subsp. *vulgare*) 100% A0A1D5TLF9 *Triticum aestivum* Uncharacterized protein 90.9%	Biological Process: GO:0006468 protein phosphorylation, GO:0007166 cell surface receptor signaling pathway Molecular Function: GO:0004672 protein kinase activity, GO:0005524 ATP binding Cellular component: -	-
ABS/RC ET_o_/RC TRo/RC WUE	3256392	barke_contig_370440|2|5.38|6729480	77: 29(1), 5(1/2), 24(2), 19(3)	F2DHH6 Predicted protein (*Hordeum vulgare* subsp. *vulgare*) 100%, A0A1D5TLF8 Uncharacterized protein (*Triticum aestivum*) 71.4%	Biological Process: GO:0006468 protein phosphorylation, GO:0007166 cell surface receptor signaling pathway Molecular Function: GO:0004672 protein kinase activity, GO:0005524 ATP binding Cellular component: -	-
ABS/RC ET_o_/RC TR_o_/RC WUE	3263978	-	20: 5(1), 8(1/2), 7(2), 0(3)	-	Biological Process: GO:0055114 oxidation-reduction process Molecular Function: GO:0005506 iron ion binding, GO:0016705 oxidoreductase activity, acting on paired donors, with incorporation or reduction of molecular oxygen, GO:0020037 heme binding Cellular component: -	-
ET_o_/RC RWC TR_o_/RC	3266919	morex_contig_43792|2|10.87|15208600	53: 12(1), 10(1/2), 16(2), 15(3)	M8CEN7 F-box/WD-40 repeat-containing protein (*Aegilops tauschii*) 87.5%	Biological Process: - Molecular Function: GO:0003676 nucleic acid binding, GO:0005515 protein binding Cellular component: -	-
ABS/RC ET_o_/RC TR_o_/RC WUE	3267465	bowman_contig_10198|2|5.84|7125040	19: 6(1), 7(1/2), 6(2), 0(3)	A0A287GXP9 Uncharacterized protein (*Hordeum vulgare* subsp. *vulgare*) 100%	Biological Process: - Molecular Function: - Cellular component: -	-
ABS/RC ET_o_/RC TR_o_/RC WUE	3268244	morex_contig_135260|2|5.38|6729480	20: 5(1), 8(1/2), 7(2), 0(3)	A0A287GXC7 Uncharacterized protein (*Hordeum vulgare* subsp. *vulgare*) 90.9%	Biological Process: GO:0055114 oxidation-reduction process Molecular Function: GO:0005506 iron ion binding, GO:0016705 oxidoreductase activity, acting on paired donors, with incorporation or reduction of molecular oxygen, GO:0020037 heme binding Cellular component: -	-
RWC TR_o_/RC WUE	3268875	morex_contig_162954|2|1.13|1370200	53: 27(1), 5(1/2), 18(2), 3(3)	M8AY22 Putative disease resistance protein (*Aegilops tauschii*) 78.9%, M7YV27 Disease resistance protein RGA2 (*Triticum urartu*) 84.2%	Biological Process: - Molecular Function: GO:0043531 ADP binding Cellular component: -	-
ABS/RC ET_o_/RC TR_o_/RC	3268955	morex_contig_43044|2|2.27|3660480	36: 9(1), 10(1/2), 5(2), 12(3)	A0A287GVF5 Uncharacterized protein (*Hordeum vulgare* subsp. *vulgare*) (100%), M7ZGA7 G patch domain-containing protein 8 (*Triticum urartu*) (100%)	Biological Process: - Molecular Function: GO:0003676 nucleic acid binding Cellular component: -	-
RWC TR_o_/RC WUE	3269335	bowman_contig_845808|2|4.25|5090200	45: 22(1), 3(1/2), 17(2), 3(3)	M0W3T9 Serine/threonine-protein kinase (*Hordeum vulgare* subsp. *vulgare*) 100%	Biological Process: GO:0006468 protein phosphorylation, GO:0048544 recognition of pollen Molecular Function: GO:0004672 protein kinase activity, GO:0004674 protein serine/threonine kinase activity, GO:0005524 ATP binding Cellular component: -	-
RWC WUE	3269712	-	48: 25(1), 5(1/2), 15(2), 3(3)	A0A287GXL8 Uncharacterized protein (*Hordeum vulgare* subsp. *vulgare*) 100%	Biological Process: GO:0006468 protein phosphorylation Molecular Function: GO:0004672 protein kinase activity, GO:0005524 ATP binding Cellular component: -	-
RWC WUE	3269714	-	47: 25(1), 5(1/2), 14(2), 3(3)	A0A287GXN2 Uncharacterized protein (*Hordeum vulgare* subsp. *vulgare*) 100%	Biological Process: GO:0006468 protein phosphorylation GO:0007166 cell surface receptor signaling pathway Molecular Function: GO:0004672 protein kinase activity GO:0005524 ATP binding Cellular component: -	-
RWC WUE	3271015	bowman_contig_172445|2|5.38|6729480	44: 24(1), 4(1/2), 13(2), 3(3)	A0A287GXL8 Uncharacterized protein (*Hordeum vulgare* subsp. *vulgare*) 94.1%	Biological Process: GO:0006468 protein phosphorylation Molecular Function: GO:0004672 protein kinase activity GO:0005524 ATP binding Cellular component: -	-
RWC TR_o_/RC WUE	3271630	barke_contig_314750|2|6.06|7125040	51: 26(1), 5(1/2), 17(2), 3(3)	A0A1D5UAG0 Uncharacterized protein (*Triticum aestivum*) 86.4%	Biological process: - Molecular Function: GO:0043531 ADP binding Cellular component: -	-
ABS/RC ET_o_/RC TR_o_/RC WUE	3272114	bowman_contig_852765|2|5.38|6335160	20: 5(1), 8(1/2), 7(2), 0(3)	A0A287GVB8 Uncharacterized protein (*Hordeum vulgare* subsp. *vulgare*) 100%	Biological Process: - Molecular Function: - Cellular component: -	-
RWC WUE	3273166	bowman_contig_11541|2|2.27|1582240	51: 11(1), 9(1/2), 15(2), 16(3)	A0A287GUZ0 Uncharacterized protein (*Hordeum vulgare* subsp. *vulgare*) 100%	Biological process: - Molecular Function: GO:0016747 transferase activity, transferring acyl groups other than amino-acyl groups Cellular component: -	-
ABS/RC PI_ABS_ PI_CSm_ PI_CSo_ RC/CS_m_ RC/CS_o_ TR_o_/RC WUE	3274182	bowman_contig_860506|7|118.34|568919440	53: 24(1), 12(1/2), 17(2), 0(3)	F2EE38 Protein DETOXIFICATION (*Hordeum vulgare* subsp. *vulgare*) 100%	Biological Process: GO:0006855 drug transmembrane transport GO:0055085 transmembrane transport Molecular Function: GO:0015238 drug transmembrane transporter activity, GO:0015297 antiporter activity Cellular Component: GO:0016020 membrane	-
RWC TR_o_/RC WUE	3432939	-	49: 24(1), 5(1/2), 18(2), 2(3)	M8BTQ3 Putative disease resistance protein RGA4 (*Aegilops tauschii*) 92.3%	Biological process: - Molecular Function: GO:0043531 ADP binding Cellular component: -	-
RWC WUE	3433049	barke_contig_512673|2|2.27|4051080	46: 20(1), 5(1/2), 18(2), 3(3)	A0A287GV69 Uncharacterized protein (*Hordeum vulgare* subsp. *vulgare*) 90.9%	Biological process: - Molecular Function: GO:0043531 ADP binding Cellular component: -	-
RWC WUE	3433111	barke_contig_512673|2|2.27|4051080	49: 24(1), 4(1/2), 18(2), 3(3)	A0A287GV69 Uncharacterized protein (*Hordeum vulgare* subsp. *vulgare*) 81.8%	Biological process: - Molecular Function: GO:0043531 ADP binding Cellular component: -	-
RWC TR_o_/RC WUE	3433113	-	45: 22(1), 4(1/2), 16(2), 3(3)	F2D1Y6 Predicted protein (*Hordeum vulgare* subsp. *vulgare*) 100%	Biological Process: - Molecular Function: - Cellular component: -	-
RWC WUE	3433167	barke_contig_314750|2|6.06|7125040	51: 27(1), 5(1/2), 16(2), 3(3)	M7ZTH5 Putative disease resistance protein RGA3 (*Triticum urartu*) 81%	Biological process: - Molecular Function: GO:0043531 ADP binding Cellular component: -	-
RWC TR_o_/RC WUE	3433655	morex_contig_44233|2|2.27|3660480	47: 24(1), 5(1/2), 15(2), 3(3)	A0A287GVB8 Uncharacterized protein (*Hordeum vulgare* subsp. *vulgare*) 100%	Biological Process: GO:0055114 oxidation-reduction process Molecular Function: GO:0005506 iron ion binding, GO:0016705 oxidoreductase activity, acting on paired donors, with incorporation or reduction of molecular oxygen, GO:0020037 heme binding Cellular Component: -	-
ABS/RC PI_CSm_ PI_CSo_ RC/CS_m_ RC/CS_o_ WUE	3661780	morex_contig_104845|7|118.24|568089880	34: 17(1), 9(1/2), 8(2), 0(3)	A0A1D6BU87 Carboxypeptidase (*Triticum aestivum*) 100%	Biological Process: GO:0006508 proteolysis Molecular Function: GO:0004185 serine-type carboxypeptidase activity Cellular component: -	-
ABS/RC ET_o_/RC TR_o_/RC WUE	3662546	morex_contig_135260|2|5. 38|6729480	20: 5(1), 8(1/2), 7(2), 0(3)	A0A287GXC7 Uncharacterized protein (*Hordeum vulgare* subsp. *vulgare*) 100%	Biological Process: GO:0055114 oxidation-reduction process Molecular Function: GO:0005506 iron ion binding, GO:0016705 oxidoreductase activity, acting on paired donors, with incorporation or reduction of molecular oxygen, GO:0020037 heme binding Cellular component: -	-
RWC TR_o_/RC WUE	3662618	bowman_contig_11541|2|2.27|1582240	49: 25(1), 5(1/2), 17(2), 2(3)	A0A287GUZ0 Uncharacterized protein (*Hordeum vulgare* subsp. *vulgare*) 100%	Biological process: - Molecular Function: GO:0016747 transferase activity, transferring acyl groups other than amino-acyl groups Cellular component: -	-
ABS/RC RC/CS_m_ RC/CS_o_ RWC TR_o_/RC	3914074	barke_contig_303834|2|2.27|3660480	27: 13(1), 1(1/2), 13(2), 0(3)	A0A287GVC9 Uncharacterized protein (*Hordeum vulgare* subsp. *vulgare*) 100%	Biological process: - Molecular Function: GO:0043531 ADP binding Cellular component: -	-
ABS/RC DI_o_/CS RC/CS_m_ WUE	3985849	morex_contig_62194|7|118.34|568919440	44: 10(1), 3(1/2), 12(2), 19(3)	A0A287E7F7 Protein DETOXIFICATION (*Hordeum vulgare* subsp. *vulgare*) 100%	Biological Process: GO:0006855 drug transmembrane transport, GO:0055085 transmembrane transport Molecular Function: GO:0015238 drug transmembrane transporter activity, GO:0015297 antiporter activity Cellular Component: GO:0016020 membrane	-
ABS/RC RC/CS_m_ WUE	3987313	morex_contig_62194|7|118.34|568919440	52: 15(1), 3(1/2), 14(2), 20(3)	A0A287E7F7 Protein DETOXIFICATION (*Hordeum vulgare* subsp. *vulgare*) 100%	Biological Process: GO:0006855 drug transmembrane transport, GO:0055085 transmembrane transport Molecular Function: GO:0015238 drug transmembrane transporter activity, GO:0015297 antiporter activity Cellular Component: GO:0016020 membrane	-
RWC TR_o_/RC WUE	4188851	bowman_contig_868253|2|5.38|6729480	53: 14(1), 9(1/2), 16(2), 14(3)	A0A287GX10 Uncharacterized protein (*Hordeum vulgare* subsp. *vulgare*) 100%	Biological Process: GO:0008152 metabolic process, GO:0009058 biosynthetic process Molecular Function: GO:0003824 catalytic activity, GO:0016747 transferase activity, transferring acyl groups other than amino-acyl groups Cellular component: -	-
ET_o_/RC RWC TR_o_/RC	4191362	-	54: 14(1), 10(1/2), 14(2), 13(3)	M0Y0X4 Uncharacterized protein (*Hordeum vulgare* subsp. *vulgare*) 82.4%	Biological Process: GO:0006468 protein phosphorylation Molecular Function: GO:0004672 protein kinase activity GO:0005524 ATP binding Cellular component: -	-
RC/CS_o_ RWC TR_o_/RC	4197350	-	51: 17(1), 2(1/2), 14(2), 18(3)	A0A287PF84 Uncharacterized protein (*Hordeum vulgare* subsp. *vulgare*) 100%	Biological Process: GO:0005992 trehalose biosynthetic process Molecular Function: GO:0003824 catalytic activity Cellular component: -	-
RWC WUE	4331021	-	49: 24(1), 5(1/2), 17(2), 3(3)	A0A1D5UEH7 Uncharacterized protein (*Triticum aestivum*) 100%	Biological Process: GO:0006468 protein phosphorylation Molecular Function: GO:0004672 protein kinase activity, GO:0005524 ATP binding Cellular component: -	-
ET_o_/RC RWC TR_o_/RC	4505558	-	52: 11(1), 10(1/2), 14(2), 17(3)	M7ZWY0 Pectate lyase (*Triticum urartu*) 81.3%	Biological Process: - Molecular Function: - Cellular component: -	-
RWC WUE	5242232	bowman_contig_287124|2|5.84|7125040fd	44: 23(1), 4(1/2), 14(2), 3(3)	A0A1D5TLF8 Uncharacterized protein (*Triticum aestivum*) 100%	Biological Process: GO:0006468 protein phosphorylation, GO:0007166 cell surface receptor signaling pathway Molecular Function: GO:0004672 protein kinase activity, GO:0005524 ATP binding Cellular component: -	-
RWC WUE	5242270	morex_contig_53426|2|5. 52|6729480	45: 24(1), 5(1/2), 13(2), 3(3)	F2DX78 Predicted protein (*Hordeum vulgare* subsp. *vulgare*) 100%	Biological Process: GO:0055114 oxidation-reduction process Molecular Function: GO:0005506 iron ion binding, GO:0016705 oxidoreductase activity, acting on paired donors, with incorporation or reduction of molecular oxygen, GO:0020037 heme binding Cellular component: -	-
ET_o_/RC RWC WUE	5247108	morex_contig_53426|2|5.52|6729480	54: 13(1), 9(1/2), 17(2), 15(3)	F2DX78 Predicted protein (*Hordeum vulgare* subsp. *vulgare*) 100%	Biological Process: GO:0055114 oxidation-reduction process Molecular Function: GO:0005506 iron ion binding, GO:0016705 oxidoreductase activity, acting on paired donors, with incorporation or reduction of molecular oxygen, GO:0020037 heme binding Cellular component: -	-
RWC TR_o_/RC WUE	5248093	bowman_contig_976415|2|2.27|3660480	49: 26(1), 5(1/2), 15(2), 3(3)	A0A287LJT0 Uncharacterized protein (*Hordeum vulgare* subsp. *vulgare*) 73.7	Biological Process: GO:0006468 protein phosphorylation Molecular Function: GO:0004672 protein kinase activity, GO:0005515 protein binding, GO:0005524 ATP binding Cellular component: -	-
RWC WUE	5248421	-	47: 25(1), 4(1/2), 15(2), 3(3)	M7Z355 Putative serine/threonine-protein kinase-like protein CCR3 (*Triticum urartu*) 95.5%	Biological Process: GO:0006468 protein phosphorylation, GO:0007166 cell surface receptor signaling pathway Molecular Function: GO:0003676 nucleic acid binding, GO:0004672 protein kinase activity, GO:0005524 ATP binding, GO:0008270 zinc ion binding Cellular component: -	-
RWC WUE	5248966	-	48: 23(1), 5(1/2), 17(2), 3(3)	M0VDL0 Uncharacterized protein (*Hordeum vulgare* subsp. *vulgare*) 81.8%	Biological Process: - Molecular Function: - Cellular component: -	-
RWC WUE	6283867	morex_contig_1590255|2|5.38|6729480	57: 13(1), 10(1/2), 19(2), 15(3)	A0A1D5TLF8 Uncharacterized protein (*Triticum aestivum*) 85%	Biological Process: GO:0006468 protein phosphorylation, GO:0007166 cell surface receptor signaling pathway Molecular Function: GO:0004672 protein kinase activity, GO:0005524 ATP binding Cellular component: -	-
ABS/RC ET_o_/RC RC/CS_o_ TR_o_/RC WUE	3987113*	barke_contig_277357|2|5.42|6729480	76: 32(1), 7(1/2), 17(2), 20(3)	A0A287GXL8 Uncharacterized protein (*Hordeum vulgare* subsp. *vulgare*) 100%	Biological Process: GO:0006468 protein phosphorylation Molecular Function: GO:0004672 protein kinase activity, GO:0005524 ATP binding Cellular component: -	-
ABS/RC ET_o_/RC TR_o_/RC WUE	5259430*	barke_contig_277357|2|5.42|6729480	79: 32(1), 7(1/2), 20(2), 20(3)	A0A287GXL8 Uncharacterized protein (*Hordeum vulgare* subsp. *vulgare*) 100%	Biological Process: GO:0006468 protein phosphorylation Molecular Function: GO:0004672 protein kinase activity, GO:0005524 ATP binding Cellular component: -	-
ET_o_/RC TR_o_/RC WUE	3272114*	bowman_contig_852765|2|5.38|6335160	50: 26(1), 5(1/2), 15(2), 4(3)	A0A1D5UB81 Uncharacterized protein (*Triticum aestivum*) 95.5%	Biological Process: GO:0008152 metabolic process Molecular Function: GO:0010333 terpene synthase activity, GO:0016829 lyase activity Cellular component: -	-
ET_o_/RC TR_o_/RC WUE	3271015*	bowman_contig_172445|2|5.38|6729480	21: 6(1), 8(1/2), 7(2), 0(3)	A0A287GXL8 Uncharacterized protein (*Hordeum vulgare* subsp. *vulgare*) 94.1%	Biological Process: GO:0006468 protein phosphorylation Molecular Function: GO:0004672 protein kinase activity, GO:0005524 ATP binding Cellular component: -	-
RWC WUE	3256857*	morex_contig_80364|7|70.96|342961680	77: 17(1), 12(1/2), 28(2), 20(3)	A0A287WY92 Uncharacterized protein (*Hordeum vulgare* subsp. *vulgare*) 100%	Biological Process: GO:0006355 regulation of transcription, DNA-templated Molecular Function: GO:0003700 DNA-binding transcription factor activity, GO:0043565 sequence-specific DNA binding Cellular component: -	-
ET_o_/RC TR_o_/RC WUE	100006951*	barke_contig_327712|2|3.82|4051080	21: 6(1), 8(1/2), 7(2), 0(3)	A0A287S5F9 Uncharacterized protein (*Hordeum vulgare* subsp. *vulgare*) 94.1%	Biological Process: - Molecular Function: - Cellular component: -	-
ABS/RC PI_ABS_ RC/CS_m_ RC/CS_o_ TR_o_/RC WUE	100005723*	morex_contig_62194|7|118.34|568919440	37: 11(1), 9(1/2), 17(2), 0(3)	A0A287E7F7 Protein DETOXIFICATION (*Hordeum vulgare* subsp. *vulgare*) 100%	Biological Process: GO:0006855 drug transmembrane transport, GO:0055085 transmembrane transport Molecular Function: GO:0015238 drug transmembrane transporter activity, GO:0015297 antiporter activity Cellular Component: GO:0016020 membrane	-
ET_o_/RC TR_o_/RC WUE	3433049*	barke_contig_512673|2|2.27|4051080	39: 10(1), 9(1/2), 7(2), 13(3)	A0A287GV69 Uncharacterized protein (*Hordeum vulgare* subsp. *vulgare*) 90.9%	Biological Process: - Molecular Function: - Cellular component: -	-
ABS/RC ET_o_/RC TR_o_/RC WUE	3256062	barke_contig_370440|2|5.38|6729480	71: 21(1), 10(1/2), 26(2), 14(3)	F2DHH6 Predicted protein (*Hordeum vulgare* subsp. *vulgare*) 100%	Biological Process: GO:0006468 protein phosphorylation, GO:0007166 cell surface receptor signaling pathway Molecular Function: GO:0004672 protein kinase activity, GO:0005524 ATP binding Cellular component: -	-
RWC WC	3266749*	morex_contig_39835|2|5.38|6066080	50: 26(1), 5(1/2), 15(2), 4(3)	A0A1D6RM92 Uncharacterized protein (*Triticum aestivum*) 100%	Biological Process: - Molecular Function: - Cellular component: -	-
ET_o_/RC RWC WUE	5247108	morex_contig_53426|2|5.52|6729480	54: 22(1), 7(1/2), 17(2), 8(3)	F2DX78 Predicted protein (*Hordeum vulgare* subsp. *vulgare*) 100%”	Biological Process: GO:0055114 oxidation-reduction process Molecular Function: GO:0005506 iron ion binding, GO:0016705 oxidoreductase activity, acting on paired donors, with incorporation or reduction of molecular oxygen, GO:0020037 heme binding Cellular component: -	-
RWC TR_o_/RC WUE	5248093	bowman_contig_976415|2|2.27|3660480	49: 26(1), 5(1/2), 15(2), 3(3)	A0A287LJT0 Uncharacterized protein (*Hordeum vulgare* subsp. *vulgare*) 73.7 %	Biological Process: - Molecular Function: - Cellular component: -	-
ET_o_/RC RWC TR_o_/RC WUE	100006913*	bowman_contig_13010|2|1. 13|1370200	53: 27(1), 5(1/2), 18(2), 3(3)	A0A287GVB8 Uncharacterized protein (*Hordeum vulgare* subsp. *vulgare*) 100%	Biological Process: GO:0055114 oxidation-reduction process Molecular Function: GO:0005506 iron ion binding, GO:0016705 oxidoreductase activity, acting on paired donors, with incorporation or reduction of molecular oxygen, GO:0020037 heme binding Cellular component: -	-
RWC WUE	3434138*	barke_contig_275657|2|6.34|7689240	50: 26(1), 5(1/2), 15(2), 4(3)	A0A1D5UB81 Uncharacterized protein (*Triticum aestivum*) 100%	Biological Process: GO:0008152 metabolic process Molecular Function: GO:0010333 terpene synthase activity, GO:0016829 lyase activity Cellular component: -	-
ET_o_/RC RC/CS_o_ RWC TRo/RC WUE	3265771*	morex_contig_162954|2|1.13|1370200	51: 25(1), 5(1/2), 18(2), 3(3)	M0VDL0 Uncharacterized protein (*Hordeum vulgare* subsp. *vulgare*) 100%	Biological Process: - Molecular Function: - Cellular component: -	-
RWC WUE	3257804*	barke_contig_277850|2|2.27|1501640	52: 26(1), 5(1/2), 16(2), 5(3)	F2EE71 Predicted protein (*Hordeum vulgare* subsp. *vulgare*) 100%	Biological Process: - Molecular Function: - Cellular component: -	-
ABS/RC PI_CSm_ RC/CS_m_ RC/CS_o_ WUE	3257717*	morex_contig_1569330|7|118.34|569145120	49: 21(1), 10(1/2), 18(2), 0(3)	A0A287XS60 Uncharacterized protein (*Hordeum vulgare* subsp. *vulgare*) 100%	Biological Process: - Molecular Function: - Cellular component: -	-
RC/CS_m_ RC/CS_o_ TR_o_/RC WUE	100002230*	bowman_contig_65329|2|82.40|532262560	58: 26(1), 6(1/2), 13(2), 13(3)	F2CYU9 Predicted protein (*Hordeum vulgare* subsp. *vulgare*) 100%	Biological process: - Molecular Function: GO:0016788 hydrolase activity, acting on ester bonds Cellular component: -	-
ABS/RC ET_o_/RC RC/CS_m_ RC/CS_o_ TR_o_/RC WUE	3255892*	-	56: 35(1), 10(1/2), 11(2), 0(3)	M8A2V0 Protein FIZZY-RELATED 3 (*Triticum urartu*) 86.7%	Biological Process: GO:1904668 positive regulation of ubiquitin protein ligase activity Molecular Function: GO:0005515 protein binding, GO:0010997 anaphase-promoting complex binding, GO:0097027 ubiquitin-protein transferase activator activity Cellular component: -	-
RWC TR_o_/RC WUE	3261698*	bowman_contig_11541|2|2.27|1582240	53: 27(1), 5(1/2), 18(2), 3(3)	A0A287GUZ0 Uncharacterized protein (*Hordeum vulgare* subsp. *vulgare*) 100%	Biological process: - Molecular Function: GO:0016747 transferase activity, transferring acyl groups other than amino-acyl groups Cellular component: -	-
ABS/RC PI_ABS_ PI_CSm_ PI_CSo_ RC/CS_m_ RC/CS_o_ RWC TR_o_/RC WUE	bPb_1312	1H, pos. 11,54073	64: 33 (1), 14(1/2), 16(2), 1(3)	A0A287EGI1 Uncharacterized protein (*Hordeum vulgare* subsp. *vulgare*) 95.8%	Biological Process: GO:0006468 protein phosphorylation Molecular Function: GO:0004672 protein kinase activity, GO:0005524 ATP binding Cellular Component: -	cl21453 PKc_like super family

*SNPs are asterisked. PP—physiological parameter. Exact chromosome location of the marker with gene annotation, ‘-‘unknown, number of genotypes carrying allele “1” or “1” and “2” (SNP markers) and their subpopulation affinity (by STRUCTURE), regions of local similarity of marker sequence described by UniProt (protein ID, species, percentage of similarity [%]), Gene Ontology (GO) assigned by InterPro database, CD— conserved domain identified by NCBI Conserved Domain Database (CDD). ‘-' non-predicted. Only markers with UniProt annotations are presented. For the whole dataset see Supplementary Table S8*.

Among the 162 associations mentioned above, 67 were annotated to known sequences, of which 57 were registered in the UniProt and/or GO database (Table 6). Possible function of proteins encoded by these marker sequences may be clearly grouped. (1) Some of them may be involved in cell surface signal perception, signal transduction and broadly understood gene expression regulation. The sequence of marker 3255929 was annotated to cell wall-associated receptor kinase-like protein which may be responsible for polysaccharide signal perception. Three further markers-−3256062, 3256392 and probably 6283867—are parts of the coding sequence of the same gene encoding barley's protein (F2DHH6), which is involved in the cell surface receptor signaling pathway. The same biological process is also annotated for two additional putative barley protein kinases, encoded partially by the sequences 3269714 and 5242232. Signal recognition on the cell surface is predicted for the protein, which is barley serine/threonine-protein kinase involved in pollen recognition and is encoded in part by the sequence 3269335. Five markers, including three SNPs (3269712, 3271015; SNPs: 3987113, 5259430, 3271015) are different parts of the sequence of a protein with kinase activity (barleys A0A287GXL8). These SNP sequences were associated with WUE and some OJIP parameters, and the rest of markers with WUE and RWC. Sequence 5248421 was annotated to the *CCR3* gene from *Triticum urartu* encoding protein involved in the cell surface receptor signaling pathway and possessing both protein kinase and (according to GO) nucleic acid binding activity. Also, two further markers are parts of the sequence of protein kinases: 4191362 and 4331021. Some of the kinases mentioned before may be involved not in signal transduction but metabolic activation. Some of the identified sequences encoded transcription factors (3266919 and SNP 3256857) and others are involved in proteolysis (3661780), including ubiquination (SNP 3255892).

(2) Other proteins encoded in part by the markers considered herein are involved in oxidation-reduction processes. They have very similar activity connected to binding iron ions and acting on paired donors with incorporation or reduction of molecular oxygen. Seven marker sequences matching coding regions of four proteins (three proteins are pointed by the pairs of markers) were found (markers: 3263978, 3268244 and 3662546, 5242270 and 5247108, 3433655 and SNP 100006913). Sequences of three of them have been assigned to cytochromes P450. The general role of cytochrome P450 is detoxification and, in our study, other candidate proteins with detoxification functions were identified. Barley protein A0A287E7F7 is considered by the UniProt database as a detoxification protein and, according to GO, is involved in transmembrane transport. Three markers (3985849, 3987313, SNP 100005723) associated with WUE and different chlorophyll fluorescence parameters were found within its sequence. Additionally, marker 3274182 was annotated to different protein with the same UniProt and GO categories.

(3) The next group consists of proteins involved probably in disease resistance (3268875, 3433167) or different metabolic processes, among which some may be connected to cell walls biosynthesis and hydrolysis. Sequence 4505558 was annotated to pectate lyase (in IPK barley database it is annotated to receptor-like kinase) and SNP 100002230 was a part of the sequence of barley's protein F2CYU9 with GO molecular function of hydrolase activity, acting on ester bonds (which may hydrolyze ester bonds between ferulic acid and sugars in cell walls). Trehalose biosynthesis was annotated to marker sequence 4197350. In turn, transferase activity was annotated to two different barley proteins, encoded partially in one case by the sequence 4188851 and in the other by sequences 3662618, 3273166 and SNP 326169. Terpene synthase activity (SNP 3434138) was also related to the physiological parameters discussed here. The rest of the sequences encoded uncharacterized proteins or proteins classified by GO for molecular function as ADP binding activity.

**Figure 2:**
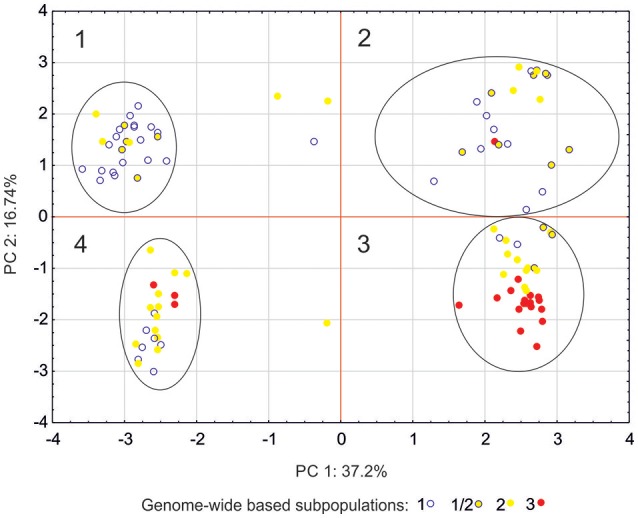
Principal component analysis (PCA) for markers associated with the studied traits and connected with the sequences identified in databases as a part of potential genes. Affinity of each genotype to one of the groupings (“candidate genes subpopulation”) can be found in Table S1.

Another approach to determine a population's structure was to use only markers associated with the studied traits and being the parts of the sequences identified in databases as parts of potential genes (candidate gene subpopulations). In this approach, the population structure revealed four subpopulations (Figure 2). This structure was different from the one observed for the studied population with the whole marker set (“genome-wide” subpopulations). Only genome-wide subpopulation 3 was over-represented in candidate gene subpopulation 3. This indicates that distribution of candidate gene alleles was independent of genomic structure of the studied barley population and independent of the origin of the lines (Table S1).

## Discussion

In the present study, the results of chlorophyll fluorescence measurements followed by OJIP testing corresponded well with leaf water status of both drought-treated and control plants of barley, but not with CO_2_ net assimilation rate. This relationship, at least in drought, may be a result of direct damaging of PSII reaction centers or other elements of the photosynthetic electron transport chain under conditions of leaf water deficit. It should be noted here that chlorophyll fluorescence parameters and CO_2_ assimilation were reported as not always closely related, especially under field or stress conditions already in the early stages of development of chlorophyll fluorescence measurement techniques (Genty et al., 1989; Edwards and Baker, 1993). In the present study, drought reduced the number of active reaction centers, while further steps for photosynthetic electron transfer were less affected. On the other hand, according to PCA analysis, *P*_N_, albeit poorly differentiated, discriminated our accessions similarly to PI_total_ in both environments, while only in the control in a way similar to quantum yield of reduction of end electron acceptors at the PSI acceptor side (ϕ_Ro_). This means that, although in both environments net assimilation rate is to some extent connected with overall photochemical efficiency, in the control it is more closely related to the activity of thylakoid electron end-acceptors.

Our study confirmed that drought-induced changes in OJIP parameters are very sensitive to changes in relative water content (RWC) in leaves. This parameter reflects the balance between water supply to the leaf tissue and transpiration rate, and thus it is considered as an important indicator of plants' water status (Schonfeld et al., 1988; Soltys-Kalina et al., 2016). Water use efficiency (WUE) is a critical measure that determines the balance between photosynthetic carbon assimilation and transpiration (Farquhar et al., 1982). In our study, in drought-treated plants WUE was negatively correlated with RWC and shared many common associations within the barley genome with RWC and OJIP parameters. The negative correlation means that plants losing less water in drought had problems with maintaining high *P*_N_, which is a typical problem the in breeding of drought tolerant crops (Lawson and Blatt, 2014; Ruggiero et al., 2017). On the other hand, in our study chlorophyll fluorescence parameters measured in drought-treated plants are not clearly connected with net assimilation rate, which is crucial for drought tolerance in the agronomic sense (Blum, 2005).

A possible explanation of this phenomenon may be suggested by the results obtained with the help of genome-wide associations (GWA). Unlike most studies using GWA to find new QTLs (He et al., 2017; Maulana et al., 2018), in our experiment we tried to identify genes in which polymorphisms of sequences associated with phenotypic traits connected with drought tolerance occur. GWA confirmed that in our population, drought-induced differences in chlorophyll fluorescence parameters were connected with water status of the leaves by the existence of some common associations with the genome. This highlights the fact that sensitivity of photosynthetic electron transport to water deficit is crucial for the observed differences between barley accessions. Markers associated in drought with both leaf water relations (RWC and WUE) and chlorophyll fluorescence parameters are correlated mostly with parameters describing the number and activities of single PSII reaction centers, while in the control the correlation is with those connected with further steps of photosynthetic electron transports. GWA is commonly used for unraveling the genetic basis of quantitative traits in populations occurring in natural conditions (Myles et al., 2009), but in our study we used this method to explain the physiological effects of drought observed in the population. For that reason, the population that we used was of a very specific kind. We selected accessions that were proven to have a very diverse response to drought—from highly tolerant to very susceptible. That explains why the difference in the number of obtained significant associations was so large for control and drought-treated plants (282 and 6,252, respectively).

Drought tolerance is a complex trait controlled by small effect genes, many of which have not yet been functionally characterized, which was also pointed out in our study (Fleury et al., 2010; Ruggiero et al., 2017). Candidate genes for leaf water relations and chlorophyll fluorescence parameters in the drought-treated plants selected herein encode proteins which are not directly involved in the control of chloroplast bioenergetics; however, they may be involved in its regulation and response to water deficit signal, with special focus to membranes and cell wall metabolism. Many of the candidate genes for RWC/WUE and chlorophyll fluorescence parameters in drought are protein kinases (e.g., F2DHH6, M0W3T9). This group of signal proteins may be involved both in the general drought stress signaling and in the regulation of stomatal control of water loss (Ruggiero et al., 2017). Special attention should be paid to kinases that may potentially act in the cell surface signal perception and/or be associated with cell walls (e.g., homolog of A0A151TV76). The former studies in barley showed that actin microfilament reorganization resulting from cell wall–plasma membrane interactions after RWC decrease are involved in ABA-independent drought signal recognition and response, including the expression of HVA1 dehydrine (Sniegowska-Swierk et al., 2016). Another candidate gene, encoding zinc finger transcription factor CCR3, is not only involved in cellular surface signal recognition but also in the control of lignin biosynthesis (Bi et al., 2011). Changes in lignin contents and composition are an important part of water loss regulation in drought (Moura et al., 2010). Candidate genes for drought response in the barley studied herein included also homologs of three multiple disease resistance genes (RGA2, RGA3, and RGA4). These proteins with homologs common in many plants are in general responsible for pathogen recognition and restriction of its growth (Liang et al., 2015). Mechanisms of this recognition is not clear but it may be connected with plasma membrane–cell wall adhesion, which has a very important function in pathogen detection and plant defense response (Underwood, 2012). Candidate gene *F2CYU9* with GO molecular function of hydrolase activity, acting on ester bonds, may hydrolyze ester bonds between ferulic acid and sugars in cell walls. This process was previously identified as important for drought tolerance in cereals (Hura et al., 2012). Moreover, in triticale the content of cell wall-bound phenolics in drought share common loci (QCWPh.4B) with some OJIP parameters (F_v_/F_m_ and ABS/CS).

Among the predicted enzymatic activities for proteins encoded in part by genes associated in our experiment with drought response in barley, trehalose synthesis was also identified. Trehalose was identified as an important element of plant drought tolerance (Fernandez et al., 2010).

Candidate genes were also found for associations common in both studied environments (drought and control). Most of them were annotated to posttranslational protein modifications or signal transduction as having protein kinases or protein binding activity, while one of the associated sequences has 100% homology with barley protein A0A287ECA encoding 3-ketoacyl-CoA synthase. The activity of these synthases is a part of log-chain fatty acid biosynthesis and in higher plants, including *Arabidopsis* and barley, they are involved in wax biosynthesis and cuticula formation (Weidenbach et al., 2014). This may explain their association with chlorophyll fluorescence parameters in drought where they strongly depend on plant water status. Neither can it be excluded that some changes in plant water status may also affect chlorophyll fluorescence in the control.

Studies of QTLs and markers, including GWAS/SNPs associated with chlorophyll fluorescence parameters, including drought treatment were performed before, show in general very complex interactions and multiple control of chlorophyll fluorescence parameters (Yin et al., 2010; Czyczyło-Mysza et al., 2011, 2013; Hao et al., 2012; Hura et al., 2017). However, direct comparison of these results with those obtained in our study is not possible because most of them were based on QTL linkage mapping using bi-parental populations, which means that most QTLs are population-specific and/or were performed with different species.

## Conclusions

Drought treatment differentiated the studied accessions of barley through revealing relationships between water status of the leaf and its photosynthetic efficiency, measured by means of chlorophyll fluorescence parameters. Variations in these characteristics, however, were not directly connected with net photosynthesis rate. Thus, chlorophyll fluorescence measurements and OJIP testing seem to be reliable tools for estimating plant water status under drought, including RWC and WUE, but not its photosynthetic activity. Additionally, pointing to the candidate genes connected with RWC, WUE and chlorophyll fluorescence parameters, our results may contribute to the physiological and molecular dissection of drought response in barley, which is the first step of understanding the complex mechanisms that control drought tolerance for future molecular breeding programs (Mir et al., 2012).

## Author Contributions

MR and JK designed the experiments. MW-J, AF, and JK conducted the experimental work. MW-J, AF, MR, and HK were responsible for data processing, bioinformatics, and statistical analysis. MR and HK interpreted the main results and drew the main conclusions. MR prepared the first version of the manuscript, but all the authors contributed in writing of the final version and approved the manuscript.

### Conflict of Interest Statement

The authors declare that the research was conducted in the absence of any commercial or financial relationships that could be construed as a potential conflict of interest.

## Supplementary Material

Supplementary Table S1 Population's structure obtained by using STRUCTURE software.

Supplementary Table S2 Statistics for phenotyping: *F*-test for comparison between phenotypic characteristics in the control and in drought; Variable contributions and principal component coordinates of cases (accessions) for PCA analysis showed in Figure 1A (control) and Figure 1B (drought).

Supplementary Table S3 Associations of DArTseq and SNP markers with physiological parameters in well-watered barley plants (control) after FDR correction.

Supplementary Table S4 Associations of DArTseq and SNP markers with physiological parameters in drought-treated barley plants after FDR correction.

Supplementary Table S5 Sequences and related data from NCBI, Gene Bank (protein ID) and IPK Barley databases for markers associated with energy trapping efficiency in PSII (F_v_/F_m_) in well-watered barley (control). Markers indicated in green are included in Table 3.

Supplementary Table S6 Sequences and related data from NCBI, Gene Bank (protein ID) and IPK Barley databases for markers associated with energy trapping efficiency in PSII (F_v_/F_m_) in barley plants under drought. Markers indicated in green are included in Table 4.

Supplementary Table S7 Sequences and related data from NCBI, Gene Bank (protein ID) and IPK Barley databases for markers associated with physiological parameters in both well-watered (control) and drought-treated barley plants. Markers indicated in green are included in Table 5.

Supplementary Table S8 Sequences and related data from NCBI, Gene Bank (protein ID) and IPK Barley databases for markers associated with at least two physiological traits in barley plants under drought with exception to the markers associated only with chlorophyll fluorescence parameters. Markers indicated in green are included in Table 6.

## Abbreviations

Chlorophyll fluorescence parameters: see Table 1
FDR: false discovery rate
GO: gene ontology
g_s_: stomatal conductance
OJIPchlorophyll fluorescence transient—the letters OJIP refer to the specific time points in the induction curve
*P*_N_: net CO_2_ assimilation rate
PSI, PSII, photosystem I and II: respectively
ROS: reactive oxygen species
RWC: relative water content (relative turgidity)
WC: water content
WUE: water use efficiency.
