# Supplementary material for: Genome-Wide Associations of Chlorophyll Fluorescence OJIP Transient Parameters Connected With Soil Drought Response in Barley
Source: Front Plant Sci. 2019 Feb 11;10:78. doi: 10.3389/fpls.2019.00078 (PMC6384533; doi:10.3389/fpls.2019.00078)
Supplement: Supplementary Table S1 — Population's structure obtained by using STRUCTURE software. [file Table_1.doc]

**Supplementary Table S1.** Population’s structure obtained by using STRUCTURE software, population’s structure on the basis of phenotype obtained by using PCA (Principal Component Analysis) and origin of the genotypes.

| **genotype** | **Subpopulation by Structure** | **Probability that the genotype belongs to subpopulation (Structure)** | | | **Subpopulation by PCA** | **Origin (crossing)** |
| --- | --- | --- | --- | --- | --- | --- |
|  |  | **1S (p)** | **2S (p)** | **3S (p)** |  |  |
| DB07022/1 | 1 | 0.717 | 0.245 | 0.038 | 1 | Bolina x DCK 128/14 |
| DB07022/11 | 1 | 0.735 | 0.201 | 0.065 | 1 | Bolina x DCK 128/14 |
| DB07080/6 | 1 | 0.464 | 0.352 | 0.184 | 1 | DCK 133/14 x Antek |
| DM4516/11 | 1 | 0.560 | 0.318 | 0.122 | 2 | Widawa x STH 367 |
| DM4690/11 | 1 | 1 | 0 | 0 | 1 | STH 9719 x Basza |
| DM4832/11 | 1 | 0.486 | 0.356 | 0.157 | 1/2 | Orthega x NAD 2800 |
| J08056/10 | 1 | 0.715 | 0.200 | 0.085 | 1 | Frontier x Pribina |
| J08084/20 | 1 | 0.537 | 0.205 | 0.257 | 2 | DCK 131/14 x Slaven |
| J08084/3 | 1 | 0.537 | 0.203 | 0.260 | 2 | DCK 131/14 x Slaven |
| J08085/10 | 1 | 0.517 | 0.376 | 0.106 | 2 | Justina x Lanfeust |
| J08085/6 | 1 | 0.647 | 0.353 | 0 | 1 | Justina x Lanfeust |
| J08086/6 | 1 | 0.565 | 0.393 | 0.043 | 2 | Justina x Slaven |
| J09003/14 | 1 | 0.567 | 0.331 | 0.102 | 2 | Eunova x Basic |
| J09008/3 | 1 | 0.660 | 0.305 | 0.035 | 1 | DCK 135/14 x Basic |
| J09011/14 | 1 | 0.615 | 0.259 | 0.126 | 3 | DCK 135/14 x Frontier |
| J09018/9 | 1 | 0.552 | 0.359 | 0.089 | 1 | Basic x Quench |
| J09038/14 | 1 | 0.666 | 0.334 | 0 | 1 | Flavour x Ella |
| OLYMPIC | 1 | 0.498 | 0.323 | 0.179 | 2 | standard (RAGT breeding) |
| SOLDO | 1 | 0.642 | 0.200 | 0.158 | 1 | standard (Nordsaat breeding) |
| STH156 | 1 | 0.878 | 0.019 | 0.103 | 1 | Suweren x KWS Atrika |
| STH240 | 1 | 1 | 0 | 0 | 4 | Suweren x Argento |
| STH293 | 1 | 0.754 | 0.246 | 0 | 2 | Iron x Suweren |
| STH330 | 1 | 1 | 0 | 0 | 1 | Ella x Suweren |
| STH33424 | 1 | 0.588 | 0.330 | 0.082 | 1 | Conchita x Basic |
| STH33425 | 1 | 0.617 | 0.382 | 0 | 1 | Conchita x Basic |
| STH366 | 1 | 1 | 0 | 0 | 4 | Ella x Suweren |
| STH373 | 1 | 0.983 | 0 | 0.017 | 4 | Ella x Suweren |
| STH391 | 1 | 1 | 0 | 0 | 1 | Ella x Suweren |
| STH393 | 1 | 1 | 0 | 0 | 1 | Ella x Suweren |
| STH464 | 1 | 0.920 | 0.079 | 0 | 4 | Fariba x Suweren |
| STH470 | 1 | 1 | 0 | 0 | 4 | Fariba x Suweren |
| STH472 | 1 | 1 | 0 | 0 | 4 | Fariba x Suweren |
| STH483 | 1 | 0.948 | 0.051 | 0 | 1 | Fariba x Suweren |
| STH491 | 1 | 0.938 | 0 | 0.062 | 3 | Fariba x Suweren |
| STH497 | 1 | 0.980 | 0 | 0.020 | 4 | Fariba x Suweren |
| Suweren | 1 | 1 | 0 | 0 | 1 | Stratus x Annabell |
| DM2682/10 | 1 | 0.625 | 0.348 | 0.028 | 3 | Philadelphia x STH 372 |
| DM2847/10 | 1 | 0.578 | 0.115 | 0.307 | 2 | Basza x Quench |
| DM2968/10 | 1 | 0.529 | 0.362 | 0.109 | 1 | Żeglarz x Prague |
| DM3494/09 | 1 | 0.686 | 0.192 | 0.123 | 2 | STH 5604 x Kirsty |
| DB07117/4 | 1/2 | 0.467 | 0.412 | 0.122 | 3 | DCK 136/14 x Blask |
| DM4188/11 | 1/2 | 0.472 | 0.465 | 0.063 | 2 | Class x MOB 9117/02 |
| DM4474/11 | 1/2 | 0.461 | 0.424 | 0.115 | 1 | Tocada x Anaconda |
| DM4479/11 | 1/2 | 0.468 | 0.477 | 0.055 | 1 | Tocada x Xanadu |
| J08005/19 | 1/2 | 0.452 | 0.386 | 0.162 | 2 | Poprad x DCK 133/14 |
| J08008/17 | 1/2 | 0.523 | 0.458 | 0.019 | 3 | Slaven x Justina |
| J08060/16 | 1/2 | 0.416 | 0.392 | 0.192 | 2 | Lanfeust x Nagradowicki |
| J08060/2 | 1/2 | 0.416 | 0.392 | 0.192 | 2 | Lanfeust x Nagradowicki |
| J08062/3 | 1/2 | 0.407 | 0.412 | 0.181 | 2 | Lanfeust x DCK 129/14 |
| J09034/10 | 1/2 | 0.496 | 0.427 | 0.077 | 1 | DCK 132/14 x DCK 135/14 |
| J09046/20 | 1/2 | 0.408 | 0.421 | 0.172 | 2 | Lanfeust x Sunshine |
| STH33846 | 1/2 | 0.400 | 0.374 | 0.226 | 1 | Sunshine x Conchita |
| STH34441 | 1/2 | 0.447 | 0.447 | 0.106 | 2 | Stratus x Iron |
| STH34959 | 1/2 | 0.375 | 0.435 | 0.190 | 3 | Oberek x Ella |
| DM3049/10 | 1/2 | 0.478 | 0.414 | 0.108 | 1 | Nagradowicki x Temperament |
| DB06145-8 | 2 | 0 | 1 | 0 | 3 | DCK 134/14 x Stratus |
| DM4480/11 | 2 | 0.425 | 0.511 | 0.064 | 4 | Tocada x Xanadu |
| J08002/10 | 2 | 0.252 | 0.581 | 0.166 | 2 | Poprad x DCK 130/14 |
| J08002/5 | 2 | 0.223 | 0.613 | 0.164 | 2 | Poprad x DCK 130/14 |
| J08055/16 | 2 | 0.374 | 0.568 | 0.058 | 2 | Nagradowicki x Lanfeust |
| J09043/3 | 2 | 0.350 | 0.615 | 0.034 | 3 | Nagradowicki x Ella |
| STH294 | 2 | 0.340 | 0.660 | 0 | 1 | Iron x Suweren |
| STH301 | 2 | 0.364 | 0.636 | 0 | 1 | Iron x Suweren |
| STH33320 | 2 | 0.303 | 0.572 | 0.125 | 1 | Conchita x Despina |
| STH33562 | 2 | 0 | 1 | 0 | 3 | Kormoran x Basic |
| STH33570 | 2 | 0.051 | 0.891 | 0.058 | 1/2 | Kormoran x Iron |
| STH33575 | 2 | 0.001 | 0.938 | 0.061 | 1/2 | Kormoran x Iron |
| STH33598 | 2 | 0.001 | 0.917 | 0.082 | 3 | Kormoran x Iron |
| STH34818 | 2 | 0.349 | 0.572 | 0.079 | 4 | Skald x KWS Olof |
| STH34819 | 2 | 0.310 | 0.690 | 0 | 4 | Skald x KWS Olof |
| STH34821 | 2 | 0.252 | 0.678 | 0.071 | 4 | Skald x KWS Olof |
| STH34822 | 2 | 0.113 | 0.683 | 0.204 | 3/4 | Skald x KWS Olof |
| STH34838 | 2 | 0.381 | 0.477 | 0.142 | 4 | Skald x KWS Olof |
| STH34839 | 2 | 0.175 | 0.808 | 0.018 | 4 | Skald x KWS Olof |
| STH34851 | 2 | 0.128 | 0.822 | 0.050 | 4 | Skald x KWS Olof |
| STH34874 | 2 | 0 | 1 | 0 | 3 | Skald x Iron |
| STH34875 | 2 | 0 | 1 | 0 | 3 | Skald x Iron |
| STH34876 | 2 | 0 | 1 | 0 | 3 | Skald x Iron |
| STH34878 | 2 | 0 | 1 | 0 | 3 | Skald x Iron |
| STH34880 | 2 | 0 | 0.995 | 0.005 | 3 | Skald x Iron |
| STH34883 | 2 | 0 | 1 | 0 | 4 | Skald x Iron |
| STH34903 | 2 | 0 | 1 | 0 | 4 | Skald x Iron |
| STH34935 | 2 | 0 | 1 | 0 | 4 | Skald x Iron |
| STH34936 | 2 | 0 | 1 | 0 | 4 | Skald x Iron |
| STH34942 | 2 | 0 | 1 | 0 | 4 | Skald x Iron |
| DM2495/10 | 2 | 0.366 | 0.465 | 0.169 | 2 | BKH 2045 x DMK 291/10 |
| DM2632/10 | 2 | 0.337 | 0.454 | 0.209 | 2 | Blask x NAD 30.7.02 |
| DM2685/10 | 2 | 0.162 | 0.702 | 0.137 | 3 | Tolar x MOB 10660/02 |
| DM3084/10 | 2 | 0.281 | 0.575 | 0.143 | 3 | Kirsty x Waggon |
| STH34964 | 3 | 0 | 0 | 1 | 3 | Oberek x Ella |
| STH34984 | 3 | 0 | 0 | 1 | 3 | STH 7910 x KWS Bambina |
| STH34985 | 3 | 0 | 0 | 1 | 3 | STH 7910 x KWS Bambina |
| STH34991 | 3 | 0.152 | 0.332 | 0.517 | 4 | STH 7910 x KWS Bambina |
| STH34999 | 3 | 0 | 0 | 1 | 3 | STH 7910 x KWS Bambina |
| STH35003 | 3 | 0.017 | 0.032 | 0.951 | 3 | STH 7910 x KWS Bambina |
| STH35004 | 3 | 0 | 0 | 1 | 3 | STH 7910 x KWS Bambina |
| STH35007 | 3 | 0 | 0 | 1 | 3 | STH 7910 x KWS Bambina |
| STH35010 | 3 | 0 | 0 | 1 | 3 | STH 7910 x KWS Bambina |
| STH35011 | 3 | 0 | 0 | 1 | 3 | STH 7910 x KWS Bambina |
| STH35014 | 3 | 0 | 0 | 1 | 3 | STH 7910 x KWS Bambina |
| STH35017 | 3 | 0.153 | 0.328 | 0.519 | 4 | STH 7910 x KWS Bambina |
| STH35019 | 3 | 0.132 | 0.387 | 0.481 | 4 | STH 7910 x KWS Bambina |
| STH35020 | 3 | 0.116 | 0.307 | 0.578 | 3 | STH 7910 x KWS Bambina |
| STH35021 | 3 | 0 | 0 | 1 | 3 | STH 7910 x KWS Bambina |
| STH35023 | 3 | 0 | 0 | 1 | 3 | STH 7910 x KWS Bambina |
| STH35024 | 3 | 0 | 0 | 1 | 3 | STH 7910 x KWS Bambina |
| STH35026 | 3 | 0.102 | 0.217 | 0.681 | 2 | STH 7910 x KWS Bambina |
| STH35029 | 3 | 0.088 | 0.302 | 0.610 | 3 | STH 7910 x KWS Bambina |
| STH35031 | 3 | 0.109 | 0.326 | 0.565 | 3 | STH 7910 x KWS Bambina |
